# Midterm outcomes after delayed first rib resection for acute axillosubclavian deep vein thrombosis in Paget-Schroetter syndrome

**DOI:** 10.1016/j.jvsv.2026.102622

**Published:** 2026-09-13

**Authors:** Mohammed Alkhani, Abdullah Alshehri, Mohamed Al-Saadi, Marine Bordet, Alexandre Oliny, Matthieu Arsicot, Antoine Millon, Nellie Della Schiava

**Affiliations:** Department of Vascular and Endovascular Surgery, Hospices Civils de Lyon, Louis Pradel University Hospital, Bron, France

**Keywords:** Paget Schroetter syndrome, Thoracic outlet syndrome, Catheter-directed thrombolysis

## Abstract

**Background:**

The standard treatment for axillosubclavian deep venous thrombosis (DVT) in patients with Paget-Schroetter syndrome typically includes thrombolysis, first rib resection (FRR), and, when needed, percutaneous transluminal venoplasty (PTV) or stenting. Our institution adopted a simplified approach using anticoagulation alone followed by delayed FRR (3-6 months) without thrombolysis. This study evaluates its clinical effectiveness.

**Methods:**

We conducted a retrospective cohort study of patients who underwent FRR for upper-extremity DVT in Paget-Schroetter syndrome between January 2017 and July 2024, using a prospectively maintained database. The primary end point was postoperative clinical improvement. We also analyzed patient demographics, venous involvement, hypercoagulable status, and anticoagulation duration.

**Results:**

Of 27 identified patients, eight who underwent thrombolysis were excluded. Among the 19 patients (mean age, 28.4 years; 13 males), all presented with arm swelling and pain. The subclavian vein was involved in 15 patients and the axillary vein in four. Eleven patients were involved in competitive sports. Mean preoperative anticoagulation duration was 5.9 ± 2.1 months and was discontinued postoperatively. Most patients underwent transaxillary FRR. Postoperative duplex showed complete DVT resolution in 16 patients and partial or chronic DVT in three. Four patients required intraoperative PTV for symptomatic stenosis. At a mean follow-up of 20.3 ± 22.1 months, 17 patients were symptom-free, whereas two patients required additional PTV.

**Conclusions:**

Anticoagulation followed by delayed FRR provides effective short-term symptom relief. Avoiding thrombolysis simplifies management and may reduce risks and costs while maintaining favorable outcomes. Further studies are needed to assess long-term efficacy.

**Article Highlights**

• **Type of Research:** Single-center retrospective observational study
• **Key Findings:** This study demonstrates that a simplified management algorithm for Paget-Schroetter syndrome that omits catheter-directed thrombolysis and treats with anticoagulation alone, followed by first rib resection, can be safe and yield acceptable clinical results.
• **Take Home Message:** The use of anticoagulation alone without thrombolytic therapy or mechanical thrombectomy before first rib resection for the treatment of Paget-Schroetter syndrome can result in good clinical results.

Thoracic outlet syndrome (TOS) is defined as compression of the neurovascular bundle within the thoracic outlet, resulting in clinical manifestations depending on the primarily affected structure. Thus, it can be categorized into three types: venous, arterial, and neurogenic. The venous type of TOS may present with upper-extremity deep venous thrombosis (DVT), hence the term “effort thrombosis.”^1^ This entity accounts for 5% of all TOS cases and about 1% to 4% of all DVTs.^2^^,^^3^ Effort thrombosis of the axillosubclavian vein was first described by Sir James Paget in 1875 and Von Schroetter in 1884, and later termed “Paget-Schroetter syndrome (PSS)” by Hughes in 1949.^4^

The pathophysiology of venous TOS is believed to result from repetitive overhead arm movements. A narrowed costoclavicular space predisposes patients to intermittent compression of the subclavian vein between the clavicle and the first rib, ultimately promoting venous stenosis, scarring, and thrombosis.^2^

Clinically, PSS is most often seen in young, athletic, or otherwise active individuals engaged in occupations or sports involving repetitive upper-extremity use.^5^ Presentation typically includes acute axillosubclavian DVT with upper limb edema, cyanosis, pain, and occasionally dilated chest wall veins in case of chronic thrombosis.^6^

Diagnosis of PSS relies on history and physical examination and is confirmed with imaging modalities such as duplex ultrasound study, computed tomographic venography, magnetic resonance venography, or conventional venography.^2^^,^^7^ Early recognition and intervention are critical, as favorable outcomes are associated with timely thrombus reduction and treatment of underlying venous stenosis before first rib resection (FRR).^8^

Management strategies for PSS have evolved from simple arm elevation and anticoagulation to more complex interventions, including thrombolysis or mechanical thrombectomy, FRR, and scalenectomy.^9^ By the mid-1980s, systemic thrombolysis followed by FRR had become standard, and Machleder^10^ refined this approach to include transcatheter thrombolysis, a period of anticoagulation before FRR, and percutaneous transluminal venoplasty (PTV) for residual stenosis. However, recent studies have questioned the necessity of preoperative thrombolysis, showing that anticoagulation alone before surgery can achieve similar vein patency and favorable clinical outcomes in selected patients, suggesting that simplified treatment strategies may be effective in certain cases.^9^^,^^11^ For each technique, the ultimate goal remains the same: to relieve the limb of swelling and pain at rest or during activities.

Our institution has adopted the latest simple approach; anticoagulation alone, followed by delayed FRR after 3 to 6 months without thrombolysis. In our opinion, waiting for at least 3 months before FRR after introducing anticoagulation is beneficial. For multiple reasons, anticoagulation will help lyse the thrombus, thereby reducing the risk of pulmonary embolism. It also minimizes the risk of perioperative complications due to inflammatory reactions around the acute venous thrombosis. We aim to evaluate the clinical effectiveness of this approach.

## Methods

A retrospective cohort study was conducted at our vascular and endovascular surgical unit, using electronic medical records to compile a database of patients who underwent FRR for PSS between January 2017 and July 2024. Inclusion criteria were patients with acute upper-extremity DVT attributed to PSS who were managed with initial anticoagulation followed by delayed interval FRR. Patients who underwent catheter-directed thrombolysis (CDT) before referral were excluded.

Patients’ demographics, clinical presentation, venous segment involvement, history of physical activity in the target limb, presence of hypercoagulable conditions, perioperative management, and surgical approach were recorded. Anticoagulation type and duration before and after surgery were documented. The primary end point was clinical improvement, defined as resolution of limb swelling and pain at follow-up.

Owing to the small number of patients, only descriptive statistical analysis was performed, using the Statistical Package for Social Sciences, version 26.0 (SPSS; IBM Corp). Categorical variables were reported as frequencies and percentages. Continuous variables were reported as mean and standard deviation or median (range).

The Institutional Review Ethics Board approved the study, and all patients received an information document about the study and their data, with the option to refuse participation in data collection; in accordance with French law on retrospective data collection, all data were anonymized.

## Results

Twenty-seven patients treated surgically for PSS by FRR were identified. Nineteen patients met our inclusion criteria, and eight patients were excluded due to previous thrombolysis.

Among the 19 patients in our cohort, 13 were male, and six were female. Mean cohort age was 23.8 ± 11.9 years. None of the patients presented with hypercoagulable diseases. Among the 19 patients, 11 were engaged in competitive sports. All patients presented with arm swelling and acute arm pain. All patients presented with an axillosubclavian DVT (15 subclavian and four axillary). Fifteen patients presented with right-sided DVTs (Table I) on the dominant right arm. Diagnosis was made using an upper-arm duplex, along with a computed tomography scan.

**Table I:** Patients’ characteristics

Variables	Value
N	19
Gender, male	13 (68)
Age, years	28.8 ± 12.0
Right side	15 (79)
Competitive athletes	11 (58)
Venous segment affected	
Subclavian	15 (79)
Axillary	4 (11)

Categorical data are shown as number (%).

Anticoagulation was achieved by direct oral anticoagulants (Apixaban). All patients had at least 3 months of anticoagulation. Average preoperative anticoagulation duration was 5.9 ± 2.1 months.

Operative techniques varied by patient and surgeon preferences (Table II). Fourteen patients underwent transaxillary FRR, three had supraclavicular resection, and two underwent thoracoscopic rib resection. All rib resections included a scalenectomy. The average operative time was 139.5 ± 38.4 minutes. Four patients received perioperative PTV without stenting for persistent symptomatic venous stenosis (Table III).

**Table II:** Surgical approach

Surgical approaches	N (%)
Transaxillary	14 (74)
Supraclavicular	3 (16)
Thoracoscopic	2 (10)

Categorical data are shown as number (%).

**Table III:** Perioperative surgical data

Variables	Value
Operative time, minutes	139.5 ± 38.4
Length of stay, days	2.5 ± 0.8
Perioperative complications	0
Reinterventions (subclavian PTV for symptomatic subclavian venous stenosis)	2

*PTV*, Percutaneous transluminal venoplasty.

None of the patients presented with perioperative complications. The average hospital stay was 2.5 ± 0.8 days.

Postoperatively, anticoagulation was discontinued except for patients who underwent perioperative PTV (n = 4), who received anticoagulation for 3 months after surgery.

At a mean follow-up of 20.3 ± 22.08 months, 17 patients remained symptom-free without arm swelling, whereas two patients required subclavian PTV for symptomatic subclavian venous stenosis without stenting (Table IV; Fig).

**Table IV:** Pre-/post-first rib resection and scalenectomy (FRRS) anticoagulation duration and follow-up

Variables	Value
Preoperative anticoagulation, months	5.9 ± 2.1
Perioperative PTV	4 patients (21%)
Duration of follow-up post-FRR, months	20.3 ± 22.08
Complete resolution	17 patients (89%)

*FRR*, First rib resection; *PTV*, percutaneous transluminal venoplasty.

**Fig:**
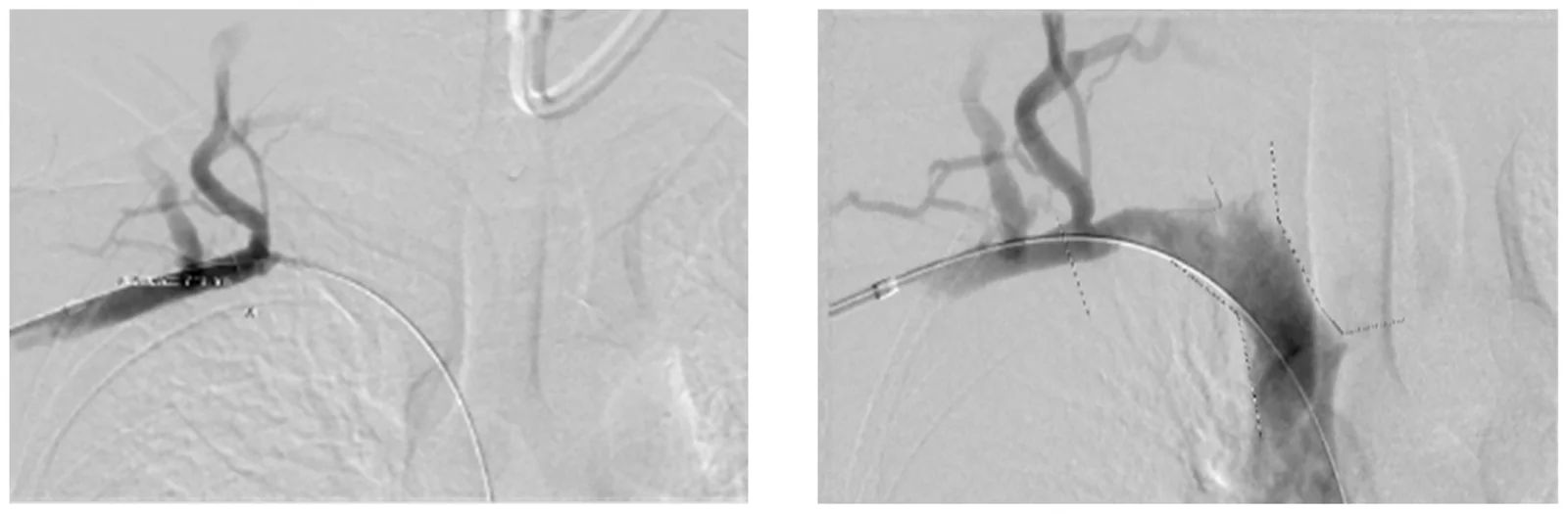
Phlebogram of a perioperative percutaneous transluminal venoplasty (PTV) of the axillosubclavian vein without the need of complementary stenting.

## Discussion

In this study, we evaluated the outcomes of managing PSS with a simplified treatment strategy consisting of anticoagulation alone followed by delayed FRR, without preoperative thrombolysis or surgical reconstruction of the subclavian vein.

Our findings demonstrate that this approach achieved excellent symptom resolution in most patients, with durable outcomes on follow-up. The mean age of this cohort was 28 years, range 16 to 61 years, reflecting the known predilection of the disease for young adults, and there was a modest male predominance. Laterality analysis demonstrated that two-thirds of patients had right-sided involvement, consistent with the mechanical pathophysiology of subclavian vein compression during dominant-arm use. No patient in our cohort had an underlying hypercoagulable disorder, further emphasizing the mechanical rather than systemic origin of thrombosis in PSS.

Over half of the patients (11) were competitive athletes, reinforcing the association between this condition and vigorous upper-extremity activity. This is consistent with its first description by Von Schrotter in 1899, suggesting a role for effort thrombosis in PSS.

Regarding surgical management, four patients needed intraoperative PTV for persistent venous stenosis; however, none required stenting, suggesting that venous decompression combined with anticoagulation was generally sufficient to restore flow. After a mean follow-up of over 20 months, outcomes were favorable: 17 patients were completely symptom-free, with no recurring arm swelling or pain, and only two needed secondary PTV for recurrent stenosis. PTV was necessary in these patients with ongoing symptomatic venous stenosis, but no one required stenting, supporting the durability of decompression alone. Importantly, no cases of pulmonary embolism were discovered during the follow-up.

It is important to note that during follow-up, ultrasound doppler surveillance was not systematically performed; instead, postoperative assessment was guided primarily by clinical outcomes and symptom resolution. This approach reflects a pragmatic strategy, emphasizing patient-reported recovery and functional status rather than routine imaging in asymptomatic individuals.

In general, current management strategies for PSS include anticoagulation, CDT or mechanical thrombectomy, FRR, and PTV, if indicated. These strategies have evolved substantially over time.^11^^,^^12^ In 1993, Machleder^10^ further refined the treatment algorithm, shifting the paradigm to include transcatheter thrombolysis instead of systemic thrombolysis, with a period of anticoagulation before FRR, and repeat venography with balloon venoplasty for residual stenosis. This approach of treatment was widely accepted and practiced as a standard stepwise therapy involving FRR, as up to one-third of patients may otherwise experience recurrent thrombosis despite initial anticoagulation and thrombolysis.^13^

More recent studies have questioned the necessity of preoperative thrombolysis. Guzzo et al^9^ conducted a retrospective study of PSS undergoing FRR, comparing two groups that received either CDT with anticoagulation or anticoagulation alone. They found no significant benefit of thrombolysis compared with anticoagulation alone in preserving venous patency or improving symptoms. Thrombolytic therapy also carries a well-established risk of bleeding. These results suggest that simplified treatment strategies may be equally effective in selected patients. Consistent with this, Minchu et al^11^ published a retrospective study of 20 patients with PSS treated with anticoagulation alone, followed by FRR. They reported that substituting anticoagulation alone for an interval of at least 6 weeks before FRR in patients with acute venous TOS achieved satisfactory axillosubclavian vein patency, 60% with complete thrombus resolution and 40% with residual wall thickening, and excellent clinical outcomes with all 20 patients remaining asymptomatic during short- and midterm follow-up.^11^ Our findings are consistent with these reports: despite omission of thrombolysis, nearly 90% remained symptom-free during follow-up. Importantly, none experienced recurrent thrombosis or pulmonary embolism.

When comparing our findings with the broader literature, important contrasts emerge. Chun et al^14^ reported on 100 patients with acute PSS, showing that initial thrombolysis combined with anticoagulation before FRR was associated with significantly higher final vein patency rates (92% vs 74%) compared with anticoagulation alone, with a trend toward improved functional outcomes measured by QuickDASH score. Similarly, the recent systematic review and meta-analysis by Hoexum et al,^15^ which pooled data from over 2600 patients across 60 cohort studies, used functional improvement as the primary outcome. They found that CDT followed by FRR achieved the highest proportion of symptom-free patients (96%), superior to both anticoagulation with FRR (80%) and anticoagulation alone (54%).^15^ Overall, these findings indicate that, although CDT followed by FRR has been demonstrated in pooled analyses to yield the highest rates of symptom-free outcomes, anticoagulation with delayed FRR was observed in our cohort to achieve similarly durable clinical outcomes while reducing treatment complexity.

Omitting thrombolysis from our protocol may offer several advantages. First, it avoids the potential risks associated with in situ thrombolysis, including bleeding complications. Second, it simplifies treatment by reducing the need for urgent interventional procedures, which can be resource-intensive and time-sensitive. Noting that during the coronavirus disease 2019 pandemic in 2020, the burden of the pandemic on the intensive care units did not interfere with our simplified management approach. Finally, it also reduces healthcare costs while still providing excellent clinical outcomes.

We have previously reported our institutional experience with the transaxillary approach for FRR in TOS, highlighting its safety and efficacy when aided by stable arm positioning systems, the TRIMANO Arthrex arm (Arthrex).^16^ This approach, originally described by Ross in 1966, has long been recognized for providing excellent access to the first rib and neurovascular structures while avoiding the morbidity associated with more invasive exposures.^17^ In venous pathology, the axillary approach provides surgical access to the jugular/subclavian confluence without manubriotomy. This surgical approach completely releases the subclavian vein. By adopting and refining this technique in our current series, we build on both the historical foundation laid by Ross and our previous work, further supporting the role of the transaxillary approach as a reliable and effective method in the management of PSS.^16^^,^^17^

FRR is usually necessary for the complete management of PSS. It has been in the management algorithm since the mid-1980s.^10^ The role of FRR in the management of PSS is mainly to relieve the anatomical conflict. It also minimizes the risk of recurrence.^18^ In a recent meta-analysis by Hoexum et al,^15^ it has been shown that only 54% of patients treated without FRR had good functional outcomes.

Our study has several limitations. The retrospective design and small sample size limit the strength of conclusions and generalizability. In addition, our follow-up period, although adequate for early outcomes, may not fully capture long-term recurrence rates. Future prospective multicenter studies are warranted to validate these findings and better define a simplified cost-effective approach as a modern treatment algorithm for PSS.

## Conclusions

Managing acute axillosubclavian DVT in PSS with anticoagulation alone, followed by delayed FRR, was effective in providing mid-term symptom relief. By omitting preoperative thrombolysis, this method simplifies treatment, reduces the potential risks of in-situ thrombolysis, and may lower costs while maintaining positive clinical outcomes. However, further studies are needed to assess its long-term effectiveness.

## Author Contributions

Conception and design: AA, MB, AO, MAr, ND

Analysis and interpretation: AM

Data collection: MAl, MAS

Writing the article: MAl, AA, MAS, AO

Critical revision of the article: MB, MAr, AM, ND

Final approval of the article: MAl, AA, MAS, MB, AO, MAr, AM, ND

Statistical analysis: Not applicable

Obtained funding: Not applicable

Overall responsibility: MAl

## Funding

No funding was provided.

## Declaration of generative AI and AI-assisted technologies in the writing process

Generative AI and AI-assisted technologies were NOT used in the preparation of this work.

## Data availability

Data regarding any of the subjects in the study has not been previously published unless specified. Data will be made available to the editors of the journal for review or query upon request.

## Disclosures

The authors declare that they have no known competing financial interests or personal relationships that could have appeared to influence the work reported in this paper.
